# Thimerosal Exposure and the Role of Sulfation Chemistry and Thiol Availability in Autism

**DOI:** 10.3390/ijerph10083771

**Published:** 2013-08-20

**Authors:** Janet K. Kern, Boyd E. Haley, David A. Geier, Lisa K. Sykes, Paul G. King, Mark R. Geier

**Affiliations:** 1 Institute of Chronic Illnesses, Inc., Silver Spring, MD 20905, USA; E-Mails: davidallengeier@comcast.net (D.A.G.); mgeier@comcast.net (M.R.G.); 2 Department of Chemistry, University of Kentucky, Lexington, KY 40506, USA; E-Mail: behaley@ctiscience.com; 3 CoMeD, Inc., Silver Spring, MD 20905, USA; E-Mails: syklone5@verizon.net (L.K.S.); paulgkingphd@gmail.com (P.G.K.)

**Keywords:** thimerosal, susceptibility, sulfation, thiols, autism

## Abstract

Autism spectrum disorder (ASD) is a neurological disorder in which a significant number of the children experience a developmental regression characterized by a loss of previously acquired skills and abilities. Typically reported are losses of verbal, nonverbal, and social abilities. Several recent studies suggest that children diagnosed with an ASD have abnormal sulfation chemistry, limited thiol availability, and decreased glutathione (GSH) reserve capacity, resulting in a compromised oxidation/reduction (redox) and detoxification capacity. Research indicates that the availability of thiols, particularly GSH, can influence the effects of thimerosal (TM) and other mercury (Hg) compounds. TM is an organomercurial compound (49.55% Hg by weight) that has been, and continues to be, used as a preservative in many childhood vaccines, particularly in developing countries. Thiol-modulating mechanisms affecting the cytotoxicity of TM have been identified. Importantly, the emergence of ASD symptoms post-6 months of age temporally follows the administration of many childhood vaccines. The purpose of the present critical review is provide mechanistic insight regarding how limited thiol availability, abnormal sulfation chemistry, and decreased GSH reserve capacity in children with an ASD could make them more susceptible to the toxic effects of TM routinely administered as part of mandated childhood immunization schedules.

## 1. Introduction

Autism spectrum disorder (ASD) is defined by qualitative impairments in social interaction, qualitative impairments in communication, and restricted and stereotyped patterns of behavior, interests, and activities [1]. Although an ASD diagnosis is defined by these three core features, recent investigations have described many health, physical, or behavioral co-morbid conditions associated with an ASD. For example, children diagnosed with an ASD were found to be more likely to have headaches/migraines, respiratory and food allergies [2], and infections [3] than typically developing children. Geier *et al.* [4] have identified other physical symptoms in children with ASD, such as gastrointestinal disturbances, incontinence, sleep problems, eating disorders, and sensory processing issues. Two significant co-morbidities associated with ASD are intellectual disabilities and epilepsy [5,6]. If autism is understood not only as a diagnosis governed by psychological criteria but also physical symptoms, this suggests that there are metabolic biomarkers and predisposing factors that can be identified and consistently associated with ASD.

A significant number of children with an ASD experience a developmental regression characterized by a loss of previously acquired skills and abilities [7,8]. Many parents report that their child was developmentally normal until sometime after birth, typically 15–24 months, at which time the child began to regress or deteriorate [9,10,11,12]. Typically reported are losses of verbal, nonverbal, and social abilities [8,10,11,12,13,14]. The reported incidence of regression in autism varies in different studies from 15% to 62% of the cases studied [7,8,13,14,15,16]. It is apparent that children diagnosed with an ASD generally divide into three groups: an early onset group, a regression group, and a heterogeneous, mixed group [8]. In addition, some children with ASD improve (to varying degrees) after regression (with and without intervention) [17].

Several studies have sought to objectively evaluate the phenomenon of autistic regression early in life. For example, Werner and Dawson [18] evaluated home videotapes of children with autism between their first and second birthday parties, with and without a reported history of regression, as well as videotapes of typically developing children. Analyses revealed that infants diagnosed with an ASD characterized by regression show similar use of joint attention and more frequent use of words and babble compared with typical infants at 12 months of age. In contrast, infants diagnosed with an ASD characterized by early onset of symptoms and no regression displayed fewer joint attention and communicative behaviors at 12 months of age. By 24 months of age, both groups of toddlers diagnosed with an ASD displayed fewer instances of word use, vocalizations, declarative pointing, social gaze, and orienting to name as compared with typically developing 24-month-olds. 

Similarly, Ozonoff *et al.* [19], in a prospective longitudinal study, evaluated the emergence of the early behavioral signs used to make an ASD diagnosis, including gaze to faces, social smiles, and directed vocalization, coded from video and rated by examiners evaluating study subjects at 6, 12, 18, 24, and 36 months of age. These investigators observed that the frequency of gaze to faces, shared smiles, and vocalizations to others were highly comparable between groups at 6 months of age, but in the group later diagnosed with an ASD, significantly declining trajectories were apparent over time. Group differences were significant by 12 months of age on most variables. These investigators concluded that their results suggest that behavioral signs of ASD are not present at birth, as once suggested by Kanner, but rather emerge over time through a process of diminishment of key social communication behaviors, and that more children than previously thought may present with a regressive course.

Although the reasons for the regression in ASD remain controversial, anecdotal reports and one study [13] suggest that the majority of parents of children diagnosed with an ASD who have experienced a regression, say that their child regressed following vaccinations. Goldberg *et al.* [13] found that the event mentioned by the majority of parents (67.6%) as concurrent with loss of skills was immunization. From a more objective point of view, it is clear that the emergence of ASD symptoms post-6 months of age, as described in the aforementioned section, follows the administration of many childhood vaccines temporally, given in accordance with the immunization schedule during the first six months of life.

Several studies suggest that children diagnosed with an ASD have abnormal sulfation chemistry, limited thiol availability, and decreased glutathione (GSH) reserve capacity, with a resulting and subsequent compromised oxidation/reduction (redox) and detoxification capacity [20,21,22,23]. For individuals who are thus compromised in regard to detoxification and/or redox, there is an increased and explicable vulnerability to brain insult.

The purpose of the present critical review is to provide mechanistic insight regarding how limited thiol availability, abnormal sulfation chemistry, and decreased GSH reserve capacity in children diagnosed with an ASD, particularly those who show evidence of regression, could make them more susceptible to the toxic effects of thimerosal (TM), the mercury (Hg)-based compound used as a preservative in many childhood vaccines, past and present. This review begins with an overview of the use of TM in vaccines.

## 2. Research Evidence

### 2.1. Use of TM in Vaccines

TM (sodium ethyl-Hg thiosalicylate, C_9_H_9_HgNaO_2_S), an organomercurial compound (49.55% Hg by weight), has been, and continues to be, used as a preservative in many childhood vaccines. For example, in the United States until the early 2000s, all of the tetanus-containing vaccines (e.g., the diphtheria-tetanus-pertussis (DTP), diphtheria-tetanus (DT), tetanus toxoid (TT), and diphtheria-tetanus-acellular-pertussis (DTaP)), and, as they were approved, the hepatitis B (HepB), Haemophilus influenza type b (Hib), and meningococcal meningitis A, C, Y, and W-135 vaccines were preserved with TM, most at a level of 0.01% TM. Then, as they were approved [24], reduced-TM formulations and finally, no-TM formulations began to displace the TM-preserved formulations, but the TM-preserved formulations were not withdrawn from the market.

With an increasing supply of reduced-TM and no-TM vaccines on the market, the expectation was that total exposure to TM would decrease sharply; however, this assumption proved to be inaccurate due to changes in the recommendations for immunization. Starting in April of 2002, the Centers for Disease Control (CDC) made a recommendation that flu shots be given to infants 6-to-23 months of age, when the only approved influenza vaccine for that age group was Sanofi Pasteur’s Fluzone^®^ which was preserved with TM [25]. In April 2002, the CDC reiterated its recommendation that pregnant women in their second and third trimesters be given a flu shot, again when all flu shots were TM preserved.

Additionally, through 2010, the CDC progressively widened the age range for annual flu vaccination until, effectively, young children were recommended to get two doses of flu vaccine initially and then receive an additional dose annually for the rest of their lives. In regard to pregnant women, CDC also removed the “second-and-third-trimester” restriction on flu shots [26,27,28].

Thus, even though reduced-TM and no-TM formulations were eventually approved by the FDA, exposure to TM through vaccination has remained widespread in the US. In 2013, more than half of all influenza vaccine doses are still TM-preserved. The net effect has been that, on average, lifetime Hg exposure from vaccines has actually increased compared to the lifetime exposure that a vaccinated person would have received under the CDC’s pre-2000 recommended vaccination schedule. Estimates are that the maximum lifetime exposure to TM a vaccinated person may receive is now more than double what it would have been had the pre-2000 vaccination schedule been maintained. Presently, in the United States, TM also remains a component in some other FDA-approved vaccine formulations including one DT and DTaP formulation, one multi-dose meningococcal meningitis vaccine, and a multi-dose TT vaccine [29]. Therefore, on average, there has been no significant decrease of TM exposure in vaccine-schedule-compliant children in the USA.

Likewise, prenatal exposure to Hg via vaccines continues to occur through the influenza vaccine still administered to pregnant women that was first recommended by the CDC in 1997 [30], since many of the influenza vaccines still, to date, contain TM [31,32]. The amount of Hg present in vaccines containing TM as a preservative nominally ranges from 12.5 μg Hg to 25 μg Hg per dose (with some vaccines containing > 25 μg Hg per dose) [29].

Worldwide, particularly in developing countries, TM is still present at preservative levels in many of the childhood vaccines such as HepB, Hib, DTP, DTwP-HepB-Hib vaccine, and various influenza vaccines [33,34,35]. Recently, the United Nations Environment Programme debated banning Hg from vaccines as part of its legal globally-binding instrument on Hg. The issues of a double standard in vaccine safety for developing countries and of access to Hg-free pharmaceuticals as a human right were highlighted by non-governmental organizations opposing the use of TM in vaccines. Many infants, particularly those in the developing world, immunized according to the recommended childhood vaccine schedule, receive about 200 μg of Hg from TM-containing vaccines during the first 6 months of life. So the cumulative dose from TM is greater in developing countries than in developed countries such as the United Kingdom, Russia, Norway, Denmark, and Sweden which have significantly restricted the use of TM. It is important to note that in 1999, the American Academy of Pediatrics and the Public Health Service called for the complete removal of TM from all vaccines [36]. 

### 2.2. TM as a Toxin

TM is not found in Nature. TM is a “designer” Hg compound in the sense that it was created and produced by humans. It was developed in 1927, to be a highly water-soluble form of Hg (metallic Hg and most ethyl-Hg (Et-Hg) compounds are not very soluble in water or water-based (aqueous) solutions) and to serve as an antimicrobial [37]. When TM is injected into a human as part of a vaccine matrix, because it is water soluble, some of the body’s most significant natural Hg-defense mechanisms are bypassed. Though TM is rapidly metabolized into various Et-Hg species (including mainly Et-Hg chloride and some Et-Hg hydroxide) in aqueous solutions and bodily fluids, its degradation does not detoxify the Hg because the end-point metabolites are tissue–retained Hg^2+^ species that are still toxic to the tissues that retain them. This is the case because all forms of Hg: elemental, inorganic and organic, are toxic to human physiology [38]. Of the toxic non-radioactive metals, Hg is the most toxic, even more toxic than lead to human fetal and neuronal cells [39,40].

In the human body, TM is broken down into Hg^2+^ species that tightly bind with the sulfur (S) residues in cellular components such as enzymes, organelles, cytoskeleton, and membranes that are critical to normal cell function [41]. Many times, the sulfhydryl (-SH) group of the amino acid l-cysteine (Cys) is the active site or is an important functional site of the protein molecule. Thus, when the Hg^2+^ species bind with enzymes, proteins, ion channels, membranes, *etc*., they alter normal cellular function and, in many instances, render the enzymes, proteins, ion channels, membranes, *etc*., essentially nonfunctional. In addition, the Hg^2+^ species present within tissues tend to bioaccummulate, especially in the brain (it is energetically very difficult for Hg^2+^ species to cross the blood-brain-barrier to back into the body), and inhibit the intracellular production and recycling of the oxidized GSH to reduced GSH [42].

Because the breakdown of TM in the body produces mainly Et-Hg chloride, which is fat soluble, it and other similar alkyl Hg compounds can pass through the brain membrane [43,44]. Once this Et-Hg compound enters the brain, it is metabolized, finally becoming tissue-retained inorganic Hg (Hg^2+^) species. Thus, to a large degree, TM is degraded into long-retained Hg^2+^ species [45].

For example, after a TM-solution was injected into rats in amounts that mimic human vaccine exposure, Rodrigues *et al.* [46], found that five days after exposure, the total Hg in the brain was present as a mixture of Hg^2+^ species (about 63%), Et-Hg species (13.5%) and, unexpectedly, Me-Hg species (23.7%), while only a low level of Hg^2+^ species were found in the rat’s blood. Studies indicate that, once de-alkylated, the resulting tissue-retained Hg^2+^ species can remain in the brain from several years to decades following exposure [47]. Its toxic effects also last for the duration.

A recent study postulates that the membrane potential of cells and mitochondria can cause the intracellular levels and the intra-mitochondrial levels of the Et-Hg species to be between 5.6 and 1,000 times the plasma levels, respectively [43]. In addition, research on endothelial cell membranes shows that TM, as well as Hg^2+^ and Me-Hg compounds, induce breakdown of membrane integrity, leading to leaky membranes. Associated with this breakdown is a major loss of cellular GSH levels [48]. Studies show that the intestinal epithelial membrane is similarly affected by these toxins [49]. Leakage from such complex cell membranes indicates that many of the toxic effects of TM, Me-Hg, and Hg^2+^ may be secondary to the induction of leaky membranes. For example, antibodies to foods may be due to food peptides leaking from the intestines into the blood where they are perceived as foreign bodies. Such an association would explain the high rates of food allergies among children diagnosed with an ASD.

### 2.3. TM Decreases GSH and Thiol Levels in General

Thiols are compounds which contain the thiol group (-SH) attached to a carbon atom. Examples of common biochemical thiols are Cys, N-acetylcysteine (NAC), and GSH. Metallothioneins (MTs) are protein thiols [50]. TM acts as a -SH inhibitor [51]. 

Many studies show that TM decreases GSH availability in human [52] and animal cells [48,53]. James *et al.* [52], for example, showed that TM caused the depletion of intracellular GSH in human neuroblastoma and glioblastoma cell lines, and Agrawal *et al.* [54] found that TM reduces GSH in human dentritic cells. In animals, for instance, Abdel-Rahman *et al.* [55] found that TM reduces brain levels of GSH in adult mice and that it persisted for many weeks following exposure.

Me-Hg has also been found to reduce GSH levels [56] and inhibit GSH production [42]. As reported by Stringari *et al.* [56] and other studies [57,58], the GSH antioxidant system is a significant molecular target of Hg, and during the early postnatal period, Hg exposure results in decreased GSH levels and decreased activities of GSH-related enzymes [56]. Moreover, in a follow-up study, Stringari *et al.* [42] found that Hg exposure effectively inhibited the developmental profile of the cerebral GSH antioxidant system during the early postnatal period. The authors stated that the inhibition of the maturation of the GSH antioxidant system might contribute to the oxidative damage seen after prenatal Hg exposure because, even though the cerebral Hg concentration in mice decreased later in the postnatal period, the GSH levels, GSH peroxidase (GPx) and glutathione reductase (GR) activities remained decreased in the mice prenatally exposed to Hg. These authors’ findings corroborate previous reports that indicate prenatal exposure to Hg adversely affects the GSH antioxidant systems by inducing biochemical alterations which persist even after the Hg tissue levels decrease to the same levels as those found in the controls.

In addition, TM quickly reacts with other thiols forming Et-Hg adducts, reducing the availability of thiols in the cell [59]. Studies have shown that TM and other forms of Hg decrease the levels of total cellular thiols in general [60,61]. For example, Hagele *et al.* [61] described that mercuric chloride (HgCl) (inorganic form), Me-Hg chloride (an environmental-related form), and TM (the pharmaceutical form) significantly induced a decrease in the levels of total cellular thiols. Critically important is that many studies have shown that the degree of cellular damage from TM is directly related to the availability of thiols [62].

Although it is commonly understood that the effects of TM are dose dependent, thiols play a critical role in mitigating the level of toxicity from TM [63]. Thus, TM adversely impacts the very systems needed to lessen its toxic effects [59]. Because the adverse impact of TM is then also a function of the level and availability of thiols in the cells, thiol availability becomes a second variable to the toxicity equation in addition to dose:

*Exposure (Dose) + Susceptibility (Thiol Content/Availability) = Outcomes (Level of Insult)*

The following section reviews the research showing that the degree of cellular damage from TM is related to the availability of thiols.

### 2.4. TM Effects and the Importance of Thiols

Studies using tissue culture show that the availability of thiols, particularly GSH, can influence the effects of TM and other Hg compounds [59]. Thiol-modulating mechanisms affecting the cytotoxicity of TM have been shown [59]. Wu *et al.* [59], for example, who examined the interaction of TM with topoisomerase II alpha and protein and non-protein thiols and with DNA, showed that depletion of intracellular GSH with buthionine sulfoximine treatment greatly increased the toxic effects of TM in K/VP.5 cells. Makani *et al.* [64] found that TM induces apoptosis in T cells via a mitochondrial pathway which caused oxidative stress and the depletion of GSH in Jurkat T cells (human T cell lymphoblast-like cell line); however, exogenous GSH apparently intercepted the TM and protected T-cells from TM-induced apoptosis. These findings, substantiated by these and many other studies, show that Hg toxicity is dependent on the cellular content of GSH [65]. This includes both the cytotoxic and the immunotoxic effects of Hg [66].

Pretreatment with other thiols, in addition to GSH, can also reduce the toxic effects of TM [67]. Migdal *et al.* [67], for example, examined the effects of TM on human monocyte-derived dendritic cells and found that TM and Hg derivatives induced dendritic cells’ activation, as measured by CD86 and HLA-DR overexpression associated with the secretion of tumor necrosis factor alpha and interleukin 8. Importantly, they found that pre-treatment with NAC (a thiol and a reactive oxygen species scavenger) strongly decreased the chemically induced overexpression of CD86. Similarly, Mian *et al.* [68], who found that the prolonged oxidative stress caused by TM induced a remarkable cleavage of focal adhesion kinase [which is accompanied by apoptosis (cell death)], also found that these effects were almost completely blocked by the pretreatment with NAC. Anundi *et al.* [53] described a molecular mechanism by which TM exposure rapidly induced oxidative stress and subsequent cellular lysis following GSH depletion in isolated hepatocytes. Importantly, they found that the addition of Cys could reverse the cellular toxicity of TM.

Nabemoto *et al.* [60] examined the effects of TM on stimulated arachidonic acid (AA) release in rat pheochromocytoma PC12 cells. They found that TM stimulated AA release in an irreversible manner and that monothiol compounds (such as l-Cys and GSH) and dithiol compounds (such as dithiothreitol) decreased the TM effect.

One of the major toxic effects of TM is its ability to cause an increase in intracellular Ca^2+^ via Ca^2+^ influx from the extracellular space [69]. However, TM-induced Ca^2+^ influx (a secondary event following ROS induction) can be suppressed by pretreatment with NAC, but not by thiol-independent antioxidants [70]. In addition, treatment with Cys has been found to significantly decrease the reduction of the GSH content by TM and the increase in the intracellular Ca^2+^ concentration [71].

James *et al.* [52] examined cultured neuroblastoma and glioblastoma cells and found that TM-induced cytotoxicity was associated with depletion of intracellular GSH in both cell lines. Pretreatment with 100 μM GSH ethyl ester or NAC resulted in a significant increase in intracellular GSH in both cell types and prevented TM cytotoxicity. The authors went on to suggest that GSH or NAC could be considered as a possible adjunct therapy to individuals still receiving TM-containing vaccinations to prevent cytotoxicity from TM.

TM is a thiol oxidizer, and as such, thiol reducing agents such as dithiotreitol (a protective agent to prevent the oxidation of thiol groups and for reducing disulphides to dithiols) have been shown to assist in cellular protection against TM. Interestingly, using thiol-reducing compounds to reduce the effects of TM on GSH has been shown in several studies. For example, Montero *et al.* [72] found that TM caused histamine-induced Ca^2+^ release in intact HeLa cells and that this effect was reversible in the presence of dithiotreitol. Using Fura-2-loaded HeLa cells (from human cell lines), Bootman and colleagues, [73] showed that TM-evoked intracellular Ca^2+^ spikes were worsened by the depletion of GSH (by preincubation with d,l-buthionine (*S,R*)-sulfoximine) and reversed by dithiothreitol.

### 2.5. Evidence of Abnormal Sulfation Chemistry in Autism

**Scheme 1:**
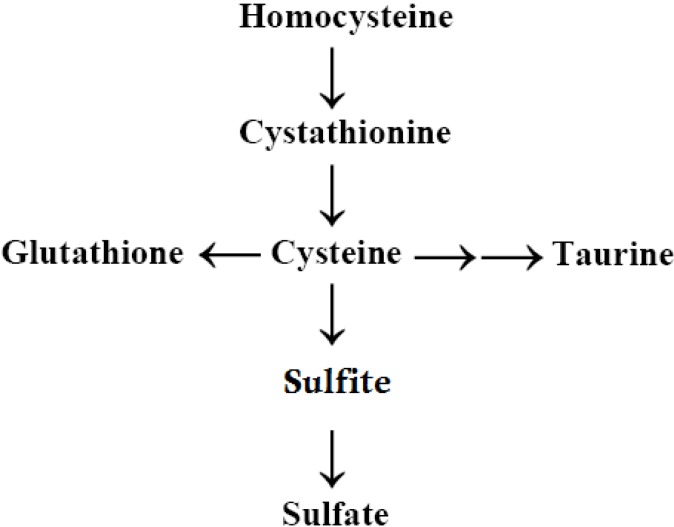
Trans-sulfuration Pathway.

Evidence for abnormal sulfation chemistry in autism began to be reported in the early 1990s, when Waring and O’Reilly [74] found low plasma levels of inorganic sulfate and sulfur oxidation deficiencies in children diagnosed with an ASD who also had food/chemical intolerances. Waring and O’Reilly found that the ratio between plasma Cys (a precursor of sulfate and taurine) and sulfate were much higher in children with autism compared to controls. In addition, this study found deficiencies in the activity of the phenol-sulfotransferase-P enzyme (PST). This enzyme requires sulfate provided by 3’-phosphoadenosine-5’-phosphosulfate, (PAPS) and catalyzes the sulfate conjugation of phenolic compounds. The authors stated that the PST enzyme itself does not appear to be lacking or genetically weakened, but that it is lacking a sufficient supply of sulfate to attach to the phenolic molecules. According to Waring and O’Reilly [74], these data may represent a fault in the production of sulfate or a problem in its being utilized at rates that exceed the speed with which cells can process Cys to sulfate (Scheme 1). Corroborating these findings, Alberti *et al.* [75] also found that children diagnosed with an ASD had low sulfation capacity.

To determine if the low plasma levels of inorganic sulfate were indicative of increased urinary sulfate loss, Waring and Klovrza [76] completed a follow-up study examining urinary levels. This follow-up study revealed that children diagnosed with an ASD excreted higher levels of urinary sulfite, sulfate, and thiosulfate but reduced levels of thiocyanate. The abnormally high levels of the first three markers suggest a dysfunction of the specific sulfate transporters, NaSi and SAT-1. These transporters move sulfate across the apical and basolateral membranes (respectively) of the renal tubule cells, and SAT-1 also functions in other organs such as the brain. In addition, there are sulfate transporters expressed in the brain, suggesting they may play a role in the transport of sulfate into the cells of the central nervous system (CNS) [77].

The reabsorption of sulfate and these other sulfur-containing molecules in the kidneys is key to maintaining body supplies of sulfur and sulfate, and this may be regulated from the gut [78]. Research by Waring and Klovrza [76] found that abnormal sulfate chemistry in children diagnosed with an ASD involved a specific population of children whose ASD symptoms manifested after 15 months of age (most were at approximately 2 years of age), with food allergies and gastrointestinal (GI) symptoms (e.g., frequent diarrhea, bloating, *etc*.). These researchers showed that these children, diagnosed with an ASD, had about 50 times the sulfite levels of the controls.

Sulfite is very neurotoxic and the inability of children diagnosed with an ASD to convert it to sulfate may be due to lack of molybdopterin caused by the displacement of molybdenum (Mo) from this cofactor by Hg. The plausibility of this occurring is evident in the structure of molybdopterin, the organometalic complex cofactor for sulfite oxidase which converts sulfite (SO^3−^) to sulfate (SO_4_^2‒^). Molybdenum is held in molybdopterin by two bonds to sulfur in the pterin and could easily be displaced by Hg^2+^, which has the highest affinity for sulfur binding. Some evidence suggests lower Mo levels in children diagnosed with an ASD [79]. In addition, Waring and Klovrza [76] reported improvements in children with autism who were given Mo supplementation. (Infants who lack molybdopterin can die of seizures early in life, which is thought to be caused by sulfite toxicity that has negative effects on white matter production.)

### 2.6. Autism, the Transsulfuration Pathway, and the Availability of Thiols

Following the work of Waring and Klovrza [76] and Waring and O’Reilly [74], many studies have continued to find abnormal sulfate levels and abnormal levels of the transsulfuration metabolites, in general, among children with ASD [21,22,80,81,82,83,84,85]. These metabolites of the transsulfuration pathway include homocysteine, cystathionine, GSH, taurine, sulfate, and Cys. In general, these transsulfuration pathway metabolites are found to be lower in children with autism than in controls. According to a review of this issue as it occurs in autism by Main *et al.* [86], the most consistent findings in the transsulfuration pathway metabolites are lower levels of plasma Cys and GSH (total and reduced). 

In a study by Geier *et al.* [84], the authors reported that not only did children diagnosed with an ASD have decreased plasma reduced GSH, plasma Cys, plasma taurine, plasma sulfate, and plasma free sulfate, but also there was also a significant inverse correlation between blood GSH levels and ASD severity using Childhood Autism Rating Scale (CARS) scores (the lower the GSH levels, the worse the autism symptoms). Similarly, Adams *et al.* [85] found significantly lower plasma GSH and plasma sulfate levels (free and total) in children with autism, and a significant inverse correlation between plasma free-sulfate levels and autism severity, meaning the lower the free-sulfate level, the greater the symptom severity. The preceding studies examined plasma or serum levels and consistently found abnormal levels of the trans-sulfuration metabolites in children diagnosed with an ASD as compared to normal controls. The following section discusses similar findings in the brains of children with an ASD.

### 2.7. Availability of GSH in the Brain of Those with Autism

Importantly, recent evidence from two studies shows that there is insufficient availability of GSH in the brains of children diagnosed with an ASD. For example, Chauhan *et al.* [87] compared DNA oxidation and GSH redox status in postmortem brain samples from the cerebellum and frontal, temporal, parietal and occipital cortex of subjects diagnosed with an ASD in comparison to age-matched normal subjects. The authors reported that levels of reduced GSH were significantly decreased and that the levels of oxidized GSH were significantly increased in the cerebellum and temporal cortex in the brain samples from the group diagnosed with an ASD, as compared to the corresponding levels in the brain samples of the controls. In other words, the brain GSH levels in those diagnosed with an ASD also appear to be inadequate.

Similarly, Rose *et al.* [88] examined frozen samples from the cerebellum and temporal cortex [Brodmann area 22 (BA22)] from individuals who had been diagnosed with an ASD and from unaffected controls. The authors found that GSH was significantly decreased in both the cerebellum and BA22 of individuals diagnosed with an ASD. In addition, 8-oxodeoxyguanosine (8-oxo-dG), a biomarker of oxidative stress and neurodegeneration, was significantly increased in the cerebellum and BA22 of those diagnosed with an ASD.

### 2.8. GSH: Complex Synthesis Process and the Potential for Excessive Demand

It is important to note that the production of GSH in the neuron requires a complex process which begins in the liver. GSH is first produced in the liver and then once it is released into the plasma it is converted to Cys and then cystine. Cystine can cross the blood-brain barrier and is taken up by the astrocyte cell [52]. The astrocyte converts the cystine to Cys and then to GSH which, once released into the extracellular space is converted to Cys [52]. The Cys is taken up by the neuron and converted to GSH. The neuron is dependent on this glial Cys for GSH synthesis [89].

GSH has so many roles, that it has the potential to be depleted due to demand. Key among these roles and functions is detoxification of xenobiotics (such as toxic metals). It is also an exogenous antioxidant that neutralizes free radicals and reactive oxygen species and is responsible for the maintenance of intracellular redox balance [86]. GSH is critical for the regulation, response, and maintenance of the immune system [90], and it modulates the effect of inflammatory cytokines [91]. GSH is also necessary for maintaining gastrointestinal integrity and for the regulation of cell proliferation [92]. GSH is also needed for the regeneration of other antioxidants such as vitamins C and E [92,93,94]. Subsequently, the lack of availability of GSH due to inadequate production or excessive demand could have an effect on many physiological systems.

As GSH production begins in the liver, it may be important to note that Et-Hg accumulates in the liver. For example, a study in rats and monkeys using injection/infusion of radiolabelled (^203^Hg) Et-Hg chloride solutions at solution levels below 1 ppm of Hg showed significant bioaccumulation in the test animals’ organs, especially the kidneys, brain, heart, and liver [95]. 

### 2.9. Direct Evidence of Decreased GSH Reserve Capacity and Increased Susceptibility in Autism

The preceding studies consistently reveal that the transsulfuration metabolites are decreased in individuals diagnosed with an ASD as compared to controls, both in blood and in the brain. The low GSH and sulfate levels found in children with ASD [21,22,23,76,80,81,82,83,84,85] predispose these children to a greater susceptibility of a brain insult because many of these transsulfuration metabolites are critically important for detoxification [20,94,96,97] and redox [98]. Although the above mentioned studies are consistent and numerous, this evidence showing an increased vulnerability to TM in children diagnosed with an ASD is still indirect.

However, a recent study found direct cellular evidence of susceptibility to TM toxicity in individuals diagnosed with an ASD. James *et al.* [23] examined lymphoblastoid cells (LCLs) derived from children diagnosed with an ASD and from unaffected controls, to assess relative concentrations of reduced GSH and oxidized GSH in cell extracts and isolated mitochondria as a measure of intracellular redox capacity [23]. The authors reported that the reduced GSH to oxidized GSH redox ratio was decreased and the percentage of oxidized GSH increased, in both cytosol and mitochondria in the LCLs from those diagnosed with autism, and that TM resulted in a greater decrease in the reduced GSH to oxidized GSH ratio and an increase in free radical generation, among cells from those diagnosed with an ASD as compared to the cells from the controls. In addition, acute exposure to physiological levels of nitric oxide (NO) decreased mitochondrial membrane potential to a greater extent in the LCLs from those diagnosed with an ASD, even though the reduced GSH to oxidized GSH ratios and ATP concentrations were similarly decreased in both cell lines. The authors concluded that the results suggest that the LCLs from those diagnosed with an ASD have a reduced GSH reserve capacity in both cytosol and mitochondria which may compromise antioxidant defense and detoxification capacity under pro-oxidant conditions. A limitation of the study is that LCLs comprise only B lymphocytes which are grown in supra-physiological concentrations of nutrients.

However, in addition to this work, the recent Sharpe *et al.* [99] study has particular relevance for this topic. In this study, researchers examined the action of low levels, ≤1,000 nM, of TM on immortalized B-cells taken from: ASD subjects, their fraternal twins, a sibling, and an age/sex matched control. They studied the effects of TM on cell proliferation and mitochondrial function from the B-lymphocytes. Eleven families were examined and compared to matched controls. They found that a subpopulation of eight individuals (four ASD, two twins, and two siblings) from four of the families showed TM hypersensitivity, whereas none of the control individuals displayed this response. Importantly, the amount of TM needed to inhibit cell proliferation in these individuals was only 40% of that required in controls. Cells hypersensitive to TM also had higher levels of oxidative stress markers, protein carbonyls, and oxidant generation. They also showed that in these hypersensitive cells, mitochondria are the target organelle conferring TM sensitivity.

### 2.10. TM as a Source of Hg Exposure in Infants and Children

There are many sources of Hg, and it is hard to apportion the contribution of TM as compared to other sources of Hg in individuals diagnosed with an ASD, particularly when considering Hg in the brain. However, there is research that shows vaccinated infants are exposed to a significant amount of TM. It is estimated that approximately 50% of the Hg exposure such infants receive comes from the periodic bolus doses of TM in certain vaccines [100]. Bigham and Copes [100] estimated a cumulative exposure of about 164 μg of dietary Hg (from breast milk) in the first 6 months of life and a cumulative dose of Et-Hg (from TM-preserved vaccines) exceeding 187.5 μg in the first 6 months of life.

These findings are in accordance with findings by Dórea *et al.* [101,102]. Dórea *et al.* [101] examined Et- and Me-Hg in hair samples of breastfed infants who had received the recommended schedule of TM-containing vaccines. They found a statistically significant inverse association between hair-Et-Hg concentrations and the time elapsed since the administration of the last TM-containing vaccine. Then, in 2012, they found that neurological development at six months was negatively associated with exposure to additional TM [102]. However, there were no developmental differences noted at 36 months.

Importantly, they also found that levels for Et-Hg species in the hair were comparable to the levels of Me-Hg species in hair on an orders-of-magnitude basis, helping to confirm that Et-Hg species from TM-containing vaccines are a significant source of fetal/infant Hg exposure. This finding is in agreement with what was predicted by Redwood *et al.*, [103] who examined the hair Hg toxicokinetics of TM-containing vaccines given to infants.

These findings are also in agreement with what has been noted in individuals diagnosed with an ASD. For example, investigators, El-baz *et al.* [104], who examined hair Hg levels in children diagnosed with an ASD whose ages ranged from 2 to 13 years (mean age 6.75, SD ± 3.26 years) and controls whose ages ranged from 2 to 11 years (mean age 5.53, SD ± 2.75 years), observed that Hg exposure from vaccines containing TM significantly contributed to hair Hg levels, whereas only mild non-significant increases of mean hair Hg levels were observed in subjects diagnosed with an ASD whose mothers had increasing numbers of dental amalgams during pregnancy or had increasing fish consumption during pregnancy [77]. In addition, the hair Hg levels of patients diagnosed with an ASD were higher in those patients with the lowest mentality and those with the most severe degree of autism according to the Childhood Autism Rating Scale. However, as this study reported, and Majewska *et al.* [105] have explained, the connection between absolute level of Hg in hair samples and the effects observed is a complicated issue because of the age-dependent differences in excretion of injected Hg from vaccines between those who are neurotypical and those diagnosed with an ASD.

It is important to note that the combined Hg exposure from vaccines and breast milk results in some infants receiving, as a daily average during the first year of life, more than 4.5 times the Environmental Protection Agency (EPA) daily Hg limit of 0.1 µg Hg/kg body weight/day. The Bigham and Copes’ [100] estimated dose of Hg from vaccines and environmental sources in the first six months of life is in excess of the safety limits of not only the EPA, but the Food and Drug Administration (FDA), the Centers for Disease Control (CDC), and the World Health Organization (WHO). Disturbingly, even when infant Hg exposure is within the level considered to be safe by the EPA, there is still an associated decrement in cognitive function found in the children exposed to that level of Hg [106,107,108]. Thus, the level of Hg exposure considered by the EPA to be safe has been shown to be inadequate and insufficiently protective.

The EPA limit is based on ingested Me-Hg and there are some significant differences between uptake from dietary ingestion [109] and bolus exposure by injection as well as differences in the neurotoxic effects of TM and other Et-Hg compounds when these are compared to a similar Me-Hg compound. The neurotoxic differences between these two families of alkyl-Hg compounds will be discussed further in the following section.

### 2.11. Neurotoxic Differences between Me- and Et-Hg

In the current environment where the use of TM in vaccines is a controversial topic, TM proponents suggest that a clear distinction exists between the toxic effects of the two Hg species, Me- and Et-Hg. Burbacher *et al.* [110], for example, showed several differences in the way that Me- and Et-Hg are metabolized in monkeys and concluded that Me-Hg did not make a good comparator. They stated that Et-Hg does not appear to stay in the blood as long as Me-Hg. This finding is often interpreted to mean that Et-Hg is excreted while Me-Hg accumulates or, as stated by the WHO [111], “Ethyl mercury has a blood half-life (3 to 7 days) much shorter than methyl mercury (45–60 days), and therefore, ethyl mercury is mostly excreted from the body and does not accumulate in the central nervous system.” However, Burbacher *et al.* [110] also concluded from their research that, “the safety of thimerosal drawn from blood Hg clearance data in human infants receiving vaccines may not be valid, given the significantly slower half-life of Hg in the brain as observed in the infant macaques.” Importantly, Burbacher *et al.* [110] found that there was a much higher proportion of inorganic Hg in the brain of TM-treated monkeys than in the brains of Me-Hg-treated monkeys (up to 71% *vs*. not more than 10%). When the non-detected results for a number of the monkeys force-fed the Me-Hg chloride is considered, the average inorganic Hg concentration in the brains of the TM-exposed monkeys was approximately three times that average for the Me-Hg-treated monkeys. In addition, they estimated, from the limited-duration data this study generated, that the dealkylation of Et-Hg was much more extensive (rapid) than the demethylation of Me-Hg during that same period. This is important because the half-life of inorganic Hg in the brain is much longer than the half-life of organic Hg.

The theory that Et-Hg does not accumulate because the blood levels decrease relatively quickly fails to take into account that the Et-Hg does not remain in the blood because it is being accumulated in the organs. For instance, in a study in rats and monkeys, using injection/infusion of radiolabelled (^203^Hg) Et-Hg chloride solutions at solution levels below 1 ppm of Hg, showed significant bioaccumulation in the test animals’ organs [95]. In the case of the monkey, the Hg level in the regions of the wet brain samples tested 8 days post-exposure was actually significantly higher (20% to 110% higher depending on the brain-region tested) than the dosing level.

Mentioned earlier, Rodrigues *et al.* [46] compared the distribution of Hg species in rat tissues following administration of TM and Me-Hg and found that, indeed, Hg remains longer in the blood of rats treated with Me-Hg compared to that of TM-exposed rats. However, they also found significant levels of Et-Hg species in the kidney, liver, and brain as well as, unexpectedly, Me-Hg species in the heart, kidney, liver, and brain of the TM-exposed rats after five days post exposure, even though there was no Me-Hg contamination in the TM used in this study.

A similar conclusion that Et-Hg did not accumulate was theorized based on the Pichichero *et al.* [112] study that reported Et-Hg was excreted in feces for several weeks following TM injection. However, a study in rats has shown that most of the Hg from the injection/infusion of solutions of Et-Hg chloride containing radiolabeled ^203^Hg does not rapidly clear the animal’s body. Only low excretion levels were present in the feces and the urine 8 days post dosing: no more than 15% of the dose given had cleared the rats via their urine and feces [113].

Since, studies, including Burbacher *et al.* [110], have shown that the final metabolites from the administration of Me-Hg chloride and TM are tissue-retained “inorganic Hg”, it is difficult to make a clear distinction between the toxic effects of Hg species, Me- and Et-Hg. Studies that do not focus on the tissue-retained “inorganic Hg” [47], which is the persistent bioaccumulating toxin in the brain and other organs and tissues or on the measurement of the toxic effects (symptoms and outcomes) from the dosing over an extended period of time cannot be relied upon to elucidate the comparative toxicity of Me- and Et-Hg compounds.

Studies that specifically compare neurotoxicity and brain changes resulting from Me- and Et-Hg exposure are somewhat mixed. Some studies show Me-Hg to be more neurotoxic than Et-Hg, and some studies show Et-Hg is more neurotoxic than Me-Hg [40,114]. The bulk of the research suggests that both forms of Hg are neurotoxic and negatively impact the health of the brain. Ueha-Ishibashi *et al.* [115], for example, examined the effects of TM on cerebellar neurons dissociated from 2-week-old rats, as compared to Me-Hg, and found that both agents (at 1 μM or higher levels) similarly decreased the cellular content of GSH in a concentration-dependent manner, suggesting an increase in oxidative stress. This was corroborated recently in a study by Zimmerman *et al.* [116] which evaluated GSH levels in C6 rat glioma cells after exposure to Me-Hg, Et-Hg, and their complexes, MeHg-S-Cys and EtHg-S-Cys and found that all the studied mercurials significantly reduced intracellular GSH levels at 4 h after the 30 min after exposure. No differences in GSH levels were observed between cells treated with Me- and Et-Hg. As mentioned earlier, Hagele *et al.* [61] found that Me-Hg chloride and TM significantly induced a decrease in the levels of total cellular thiols.

In regard to general brain health, both forms of Hg (Me- and Et-Hg) have been found to cause Hg-induced vasculo-toxicity and resulting reduced blood flow in the brain [117,118,119]. Both cerebral and cerebellar decreased blood flow can result from Hg exposure.

Often, it is suggested that Me-Hg is more toxic than Et-Hg because Me-Hg has active transport into cells through the l-type neutral amino acid carrier transport (LAT) system [120]. However, a recent study that examined the transport of Me- and Et-Hg in C6 rat glioma cell line showed that uptake of both forms is mediated, at least in part, through the LAT system. Moreover, according to the study investigators, the study showed that Me- and Et-Hg enter C6 cells by mechanisms other that LAT system [116].

It is important to note, however, that TM is injected, bypassing natural defense mechanisms; whereas, Me-Hg is typically taken orally by consuming fish or breast milk and thus does not bypass the natural defense mechanisms of the body [121]. Fish also often contain nutrients that can counteract Me-Hg toxicity, such as selenium [122].

There is some contradiction between governmental organizations on the relative toxicity of Me- and Et-Hg. The United States Food and Drug Administration considered Et- and Me-Hg as equivalent in its risk evaluation. They cite several studies that show TM to be toxic [123]. However, the WHO states that Me-Hg is toxic, but that Et-Hg is not toxic. Furthermore, the WHO Global Advisory Committee on Vaccine Safety (GACSV) has concluded that there is currently no evidence of Hg toxicity in infants, children, adolescents or adults (including pregnant women) exposed to TM in vaccines [111,124]. However, this conclusion is at odds with the numerous studies that have been published on TM since 1931 which found harm from TM [125].

### 2.12. TM and Neurodevelopmental Periods

Until approximately three years of age, the brain develops rapidly and is characterized by critical developmental periods. Evidence suggests that once a process of development is missed or altered, that developmental period cannot be fully recovered, leading to brain abnormalities that are brain region- and time-specific [126]. These sensitive periods of elevated activity and processes can create windows of vulnerability for the developing brain [127]. As such, the detrimental effects of environmental compounds upon the body and brain can be determined in part by developmental age [128]. Many studies in the animal model show that toxic exposure at different developmental periods can have different detrimental effects [56]. Stringari *et al.* [56], for example, investigated the critical phases where Me-Hg induced cerebellar toxicity during the suckling period in mice. Animals were treated with daily subcutaneous injections of a Me-Hg compound during four different periods (5 days each) during the early postnatal period: postnatal day (PND) 1–5, PND 6–10, PND 11–15, or PND 16–20. A control group was treated with normal saline. Researchers found that the postnatal exposure to Me-Hg during the second half of the suckling period caused the most damage.

In addition, infant and fetal tissue appears to be more vulnerable to some toxic effects of Hg than older children and adults [129]. This may be due, in part, to the availability, or lack thereof, of GSH. In the neonatal rat, for example, the main route of elimination of Me-Hg is by secreting the toxin into bile. This ability to secrete Hg into bile develops between 2 to 4 weeks of age and correlates with the increasing ability of the developing liver to secrete GSH into bile. Prior to 2 to 4 weeks of age, neonate rats are more vulnerable to the Hg toxin [130]. Based on radio-labeled (^203^Hg) studies of alkyl and aryl Hg compounds in rats, the principal route of early elimination is fecal and renal, but mostly fecal [113]. Estimated clearance values in the neonate rats are slower than in the adult rats, reflecting the known immaturity of renal function in neonates [131].

Specific to TM exposure, embryonic exposure to TM has been shown to produce lasting impairment of monoaminergic system in the rat brain. Following TM administration on embryonic day 9, serotonin and dopamine was found to be significantly increased on postnatal day 50 in the hippocampus [132].

Because the adverse impact of environmental compounds in the body and on the brain is a function of developmental times of exposure, this adds a third critical variable to the toxicity equation in addition to dose and thiol availability:

*Exposure (Dose) + Susceptibility (Thiol Content/Availability) + Neurodevelopmental Stage (Timing) = Outcomes (Level of Insult)*

### 2.13. Hg and the Brain Pathology in Autism

Many researchers have reported that the results of their research show a relationship between Hg and ASD [133,134,135,136,137,138,139,140,141,142,143,144,145,146,147,148,149,150]. As stated by Geier *et al.* [147], an ASD may be triggered in a child as a result of a combination of genetic/biochemical susceptibilities in the form of a reduced ability to excrete Hg and/or increased environmental exposure at key developmental times. Although other causes of ASD have been postulated, none can explain the myriad of ASD-associated pathologies found in the body and in the brain as well as Hg. For example, an examination of the parallels between the effects Hg intoxication on the brain and the brain pathology found in individuals diagnosed with an ASD reveals consistent parallels between the two [146]. Significantly, one of the signature effects of Hg is that it selectively targets large, long-range axons with subsequent abortive axonal sprouting (short, thin axons) and dentritic overgrowth. This leads to a loss of long-range connections and excessive short range connections, and this is what is found in autism. Many studies that examine the brain in autism report neuroinflammation with concomitant microglial/astrocytic activation, brain immune response activation, and elevated glial fibrillary acidic protein, and importantly this neuroinflammation appears to be chronic, not just acute, as well as excessive. It involves increased pro-inflammatory cytokine levels in the brain and aberrant nuclear factor kappa-light-chain-enhancer of activated B cells (NF-κB), both of which are activated by the presence of Hg in the brain. Many studies show oxidative stress and lipid peroxidation, decreased reduced GSH levels and elevated oxidized GSH, and mitochondrial dysfunction in both the brain in autism and in Hg intoxication. Parallels also include disruption in calcium homeostasis and signaling; inhibition of glutamic acid decarboxylase (GAD) activity; disruption of GABAergic and glutamatergic homeostasis; inhibition of IGF-1 and methionine synthase activity; and impaired methylation. Vascular endothelial cell dysfunction and pathological changes of the blood vessels and subsequent decreased cerebral/cerebellar blood flow is included. Loss of granule and Purkinje neurons in the cerebellum is a consistent finding in both Hg intoxication and autism. For a complete review of the research that identifies these parallels between the effects Hg intoxication on the brain and the brain pathology found in individuals diagnosed with an ASD, please see Kern *et al.* [146].

Since that comprehensive review, another parallel has been found between the brain serotonin system in autism and in Hg intoxication as well. Two recent studies by Ida-Eto *et al*. [132,151] shows that prenatal TM exposure alters that the brain’s serotonin systems. Importantly, they found that TM administered to pregnant rats caused a dramatic increase in the number of serotonergic neurons in the brains in the TM group (1.9-fold increase, *p* < 0.01 compared to control). Similarly, there is also an increase in the number of serotonergic neurons in the brain in autism. Azmitia *et al.* [152] examined serotonin (5-HT) axons that were immunoreactive to a serotonin transporter (5-HTT) antibody in a number of postmortem brains from children and adults with autism and found that stained axons at all ages studied indicated that the number of serotonin axons was increased in both pathways and terminal regions in the cortex from autism donors.

### 2.14. TM as a Risk Factor in Other Developmental Issues

When it is suggested that TM may contribute to the dramatic rise in the rate of ASD diagnoses due to the relationship between the increase in ASD rates and the increase in Hg exposure from vaccine-derived TM beginning in the late 1980s, the following question is often posed: “Then why aren’t all children injected with TM-containing vaccines affected?” Such a question does not take into account the unique constellation of factors:

*Exposure (Dose) + Susceptibility (Thiol Content/Availability) + Neurodevelopmental Stage (Timing) = Outcomes (Level of Insult)*

For each child and every exposure, there is a unique set of factors, the sum of which govern whether there will be adverse effects from the toxic exposure, and if so, how severe. The unique nature of individual exposure is made even more complex by subsequent exposures and the individual body’s ability to excrete some of that Hg prior to the next exposure, again bearing on thiol content and availability. An historic and intricate understanding of a toxic exposure and its many contributing and interacting factors, which culminate in determining outcome and adverse effects and the possible diagnosis of an ASD, is now emerging.

In addition, given the widespread exposure and the indicated toxicity of TM, an additional answer is that more children are affected than society currently realizes or counts. There is evidence to suggest that exposure to TM is a risk factor for other developmental issues besides autism. For example, in a study by Mrozek-Budzyn *et al.* [153], neonatal TM exposure was examined in 196 infants born to mothers attending prenatal clinics in the first and second trimesters of pregnancy in Krakow. Adverse effect of neonatal TM exposure was observed for the psychomotor development index (PDI) only at 12 and 24 months of age. No significant effect of neonatal TM exposure was found at 36 months of age; however, the overall deficit in the PDI attributable to neonatal TM exposure measured over the course of the three-year follow-up was significantly higher in TM group.

In addition, Thompson *et al.* [154] showed a significant association in boys between TM exposure from vaccines administered between birth and seven months of age in motor tics with an odds ratio of 2.19 (95% CI: 1.02 to 4.47) and phonic tics with an odds ratio of 2.44 (95% CI: 1.12–5.35). This link is supported by five other studies [136,155,156,157,158].

Another example of TM being associated with adverse effects that by and large are unrecognized comes from the 2009/2010 flu season. During this time, the maximum prenatal Et-Hg related exposure increased in the United States to nominally 50 μg during the 2009/2010 flu season via the recommended 2009/2010 seasonal-flu and H1N1 flu vaccines that contained a preservative level of TM (an increase of 25 μg). An estimated 43% of all pregnant women in the United States received the 2009 H1N1 influenza vaccine [159]. The unadjusted fetal-loss report rates for the three consecutive influenza seasons beginning with the 2008/2009 season were, in terms of reports per million pregnant women vaccinated: 6.8 (for the 2008/2009 season); 77.8 (for the 2009/2010 season); and 12.6 (for the 2010/2011 season) [31]. Thus, based on the data in Goldman’s paper [31], the influenza-vaccine related spontaneous abortion and stillbirth report rates in the 2009–2010 flu season (in the CDC’s Vaccine Adverse Events Reporting System [VAERS]) increased by more than 11-fold from the report rates in the previous flu season. In 2010/11, when the amount of Hg from TM returned to a maximum nominal amount of 25 μg, the fetal-loss reports rates returned to levels similar to what they were in the 2008/2009 flu season. Goldman stated that a synergistic fetal toxicity likely resulted from the administration of both the TM-preserved pandemic (2009-A-H1N1) and TM-preserved seasonal influenza vaccines to pregnant women during the 2009/2010 flu season.

Furthermore, an evaluation of 278,624 subjects (birth cohorts from 1990–1996) in the computerized medical records within the Vaccine Safety Datalink (VSD), revealed that there is an association between premature puberty and exposure to Hg from TM-containing vaccines [160]. Hg is a known endocrine disruptor [161] and scientists have expressed concern about the potential role of endocrine-disrupting chemicals in increasing trends in early puberty in girls [162]. A review of the literature on the effects of Hg on the endocrine system by Tan *et al.* [163] revealed that Hg accumulates in the endocrine system, causes changes in hormone concentrations, and interacts with sex hormones.

Even topical application of TM has been shown to have serious, even fatal, risks. Fagan *et al.* [164], for example, reported that when 0.1% TM (from merthiolate) was topically applied to 13 infants with omophaloceles, 10 of the 13 infants died. The Hg levels in their organs ranged from 65–2,700 times above the normal organ levels.

## 3. Conclusions

The interplay of TM with the abnormal sulfation chemistry and limited thiol availability and redox capacity observed in those diagnosed with an ASD is likely an integral factor in the etiology of autism. Limited thiol availability suggests vulnerability and the mechanisms for increased vulnerability to TM due to limited thiol availability have been delineated in this review. The associated behavioral and developmental outcomes found in ASD are plausible as a manifestation of Hg toxicity, since the brain is a target organ for TM’s toxic effects as well as a target organ for the bioaccumulation of the toxic, long-retained Hg species derived from injected Et-Hg compound exposures [95].

The evidence suggests that the abnormal sulfation chemistry, limited thiol availability, and decreased GSH reserve capacity could explain why the adverse effects of TM are greater in a subpopulation of children with this susceptibility and why the subsequent brain insult is more pronounced in them, as has been shown repeatedly in the animal model. Furthermore, it has recently been demonstrated that polymorphisms in glutathione-related genes modify Hg concentrations and antioxidant status in human subjects environmentally exposed to Hg [165].

With the rate of children diagnosed with an ASD in the US now exceeding 1 in 50 children [166] and the rate of children with neurodevelopmental/behavioral disorders in the US now exceeding 1 in 6 children [167], and the preceding evidence showing that there is vulnerability to TM that would not be known without extensive testing, the preponderance of the evidence indicates that TM should be removed from all vaccines.

## References

[1] American Psychiatric Association (2013). Diagnostic Criteria for Autistic Disorder. Diagnostic and Statistical Manual of Mental Disorders.

[2] Schieve L.A., Gonzalez V., Boulet S.L., Visser S.N., Rice C.E., van Naarden B.K., Boyle C.A. (2011). Concurrent medical conditions and health care use and needs among children with learning and behavioral developmental disabilities, National Health Interview Survey, 2006–2010. Res. Dev. Disabil..

[3] Atladóttir H.O., Thorsen P., Schendel D.E., Østergaard L., pLemcke S., Parner E.T. (2010). Association of hospitalization for infection in childhood with diagnosis of autism spectrum disorders, a Danish cohort study. Arch. Pediatr. Adolesc. Med..

[4] Geier D.A., Kern J.K., Geier M.R. (2012). A prospective cross-sectional cohort assessment of health, physical, and behavioral problems in autism spectrum disorders. Maedica (Buchar).

[5] Danielsson S., Gillberg I.C., Billstedt E., Gillberg C., Olsson I. (2005). Epilepsy in young adults with autism: A prospective population-based follow-up study of 120 individuals diagnosed in childhood. Epilepsia.

[6] Hyman S.E. (2011). Grouping diagnoses of mental disorders by their common risk factors. Am. J. Psychiatry.

[7] Stefanatos G.A. (2008). Regression in autistic spectrum disorders. Neuropsych. Rev..

[8] Ozonoff S., Williams B.J., Landa R. (2005). Parental report of the early development of children with regressive autism, the delays-plus-regression phenotype. Autism.

[9] Filipek P.A., Accardo P.J., Baranek G.T., Cook E.H., Dawson G., Gordon B., Gravel J.S., Johnson C.P., Kallen R.J., Levy S.E. (1999). The screening and diagnosis of autistic spectrum disorders. J. Autism Dev. Disord..

[10] Davidovitch M., Glick L., Holtzman G., Tirosh E., Safir M.P. (2000). Developmental regression in autism, maternal perception. J. Autism Dev. Disord..

[11] Tuchman R. (1996). Pervasive developmental disorder, neurologic perspective. Acta Neuropediat..

[12] Kern J.K., Miller V.S., Evans P.A., Trivedi M.H.  (2002). Efficacy of porcine secretin in children with autism and pervasive developmental disorders. J. Autism Dev. Disord..

[13] Goldberg W.A., Osann K., Filipek P.A., Laulhere T., Jarvis K., Modahl C., Flodman P., Spence M.A. (2003). Language and other regression, assessment and timing. J. Autism Dev. Disord..

[14] Malhi P., Singhi P. (2012). Regression in children with autism spectrum disorders. Indian J. Pediatr..

[15] Ji L., Chauhan V., Flory M.J., Chauhan A. (2011). Brain region-specific decrease in the activity and expression of protein kinase a in the frontal cortex of regressive autism. PLoS One.

[16] Hansen R.L., Ozonoff S., Krakowiak P., Angkustsiri K., Jones C., Deprey L.J., Le D.N., Croen L.A., Hertz-Picciotto I. (2008). Regression in autism, prevalence and associated factors in the CHARGE Study. Pediatrics.

[17] Zappella M. (2010). Autisticregression with and without EEG abnormalities followed by favourable outcome. Brain Dev..

[18] Werner E., Dawson G. (2005). Validation of the phenomenon of autistic regression using home videotapes. Arch. Gen. Psychiatry.

[19] Ozonoff S., Iosif A.M., Baguio F., Cook I.C., Hill M.M., Hutman T., Rogers S.J., Rozga A., Sangha S., Sigman M., Steinfeld M.B., Young G.S. (2010). A prospective study of the emergence of early behavioral signs of autism. J. Am. Acad. Child Adolesc. Psychiatry.

[20] Kern J.K., Waring R.H., Ramsden D.B., Grannemann B.D., Garver C.R., Trivedi M.H., Columbus F. (2004). Abnormal Sulfation Chemistry in Autism. Progress in Autism Research.

[21] James S.J., Cutler P., Melnyk S., Jernigan S., Janak L., Gaylor D.W., Neubrander J.A. (2004). Metabolic biomarkers of increased oxidative stress and impaired methylation capacity in children with autism. Am. J. Clin. Nutr..

[22] James S.J., Melnyk S., Jernigan S., Cleves M.A., Halsted C.H., Wong D.H., Cutler P., Bock K., Boris M., Bradstreet J.J. (2006). Metabolic endophenotype and related genotypes are associated with oxidative stress in children with autism. A. J. Med. Genet. B Neuropsychiatr. Genet..

[23] James S.J., Rose S., Melnyk S., Jernigan S., Blossom S., Pavliv O., Gaylor D.W. (2009). Cellular and mitochondrial glutathione redoximbalance in lymphoblastoid cells derived from children with autism. FASEB J..

[24] Food and Drug Administration. http://www.fda.gov/BiologicsBloodVaccines/DevelopmentApprovalProcess/BiologicalApprovalsbyYear/default.htm.

[25] Centers for Disease Control (2002). Prevention and Control of Influenza: Recommendations of the Advisory Committee on Immunization Practices (ACIP). MMWR.

[26] Centers for Disease Control (2004). Prevention and Control of Influenza: Recommendations of the Advisory Committee on Immunization Practices (ACIP). MMWR.

[27] Centers for Disease Control (2006). Prevention and Control of Influenza: Recommendations of the Advisory Committee on Immunization Practices (ACIP). MMWR.

[28] Centers for Disease Control (2009). Prevention and Control of Seasonal Influenza with Vaccines Recommendations of the Advisory Committee on Immunization Practices (ACIP). MMWR.

[29] John Hopkin’s School of Public Health Thimerosal Content in Some US Licensed Vaccines, 2012. http://www.vaccinesafety.edu/thi-table.htm.

[30] Centers for Disease Control (1997). Prevention and Control of Influenza: Recommendations of the Advisory Committee on Immunization Practices (ACIP). MMWR.

[31] Goldman G. (2013). Comparison of VAERS fetal-loss reports during three consecutive influenza seasons, was there a synergistic fetal toxicity associated with the two-vaccine 2009/2010 season?. Hum. Exp. Toxicol..

[32] Brown I.A., Austin D.W. (2012). Maternal transfer of mercury to the developing embryo/fetus: Is there a safe level?. Toxicol. Environ. Chem..

[33] Muñoz M.A., Katia Abarca V.K., Jiménez de la J.J., Luchslnger F.V., O’Ryan G.M., Ripoll M.E., Valenzuela B.M.T., Vergara F.R. (2007). Safety of thimerosal containing vaccines. Statement of the Consultive Committee of Immunizations on behalf of the Chilean Infectious Diseases Society. Rev. Chil. Infect..

[34] Dórea J.G. (2010). Making sense of epidemiological studies of young children exposed to thimerosal in vaccines. Clin. Chim. Acta..

[35] Dórea J.G. (2011). Integrating experimental (*in vitro* and *in vivo*) neurotoxicity studies of low-dose thimerosal relevant to vaccines. Neurochem. Res..

[36] Centers for Disease Control and Prevention (CDC) (1999). Thimerosal in vaccines: A joint statement of the American Academy of Pediatrics and the Public Health Service. MMWR..

[37] Budavari S. (1996). Thimerosal. The Merck Index.

[38] Environmental Protection Agency Mercury Compounds; Hazard Summary-Created in April 1992; Revised in January 2000. http://www.epa.gov/ttnatw01/hlthef/mercury.html.

[39] Dirilgen N. (2011). Mercury and lead, assessing the toxic effects on growth and metal accumulation by Lemna minor. Ecotoxicol. Environ. Saf..

[40] Geier D.A., King P.G., Geier M.R. (2009). Mitochondrial dysfunction, impaired oxidative-reduction activity, degeneration, and cell death in human neuronal and fetal cells induced by low-level exposure to Thimerosal and other metal compounds. Toxicol. Environ. Chem..

[41] Farina M., Avila D.S., da Rocha J.B., Aschner M. (2013). Metals, oxidative stress and neurodegeneration: A focus on iron, manganese and mercury. Neurochem. Int..

[42] Stringari J., Nunes A.K., Franco J.L., Bohrer D., Garcia S.C., Dafre A.L., Milatovic D., Souza D.O., Rocha J.B., Aschner M., Farina M. (2008). Prenatal methylmercury exposure hampers glutathione antioxidant system ontogenesis and causes long-lasting oxidative stress in the mouse brain. Toxicol. Appl. Pharmacol..

[43] Sharpe M.A., Livingston A.D., Baskin D.S. (2012). Thimerosal-derived ethylmercury is a mitochondrial toxin in human astrocytes, possible role of Fenton chemistry in the oxidation and breakage of mtDNA. J. Toxicol..

[44] Budavari S. (1996). Ethylmercuric Chloride. The Merck Index.

[45] Qvarnström J., Lambertsson L., Havarinasab S., Hultman P., Frech W. (2003). Determination of methylmercury, ethylmercury, and inorganic mercury in mouse tissues, following administration of thimerosal, by species-specific isotope dilution GC-inductively coupled plasma-MS. Anal. Chem..

[46] Rodrigues J.L., Serpeloni J.M., Batista B.L., Souza S.S., Barbosa F. (2010). Identification and distribution of mercury species in rat tissues following administration of thimerosal or methylmercury. Arch. Toxicol..

[47] Sugita M. (1978). The biological half-time of heavy metals. The existence of a third, “slowest” component. Int. Arch. Occup. Environ. Health.

[48] Secor J.D., Kotha S.R., Gurney T.O., Patel R.B., Kefauver N.R., Gupta N., Morris A.J., Haley B.E., Parinandi N.L. (2011). Novel lipid-soluble thiol-redox antioxidant and heavy metal chelator, N,N'-bis(2-mercaptoethyl)isophthalamide (NBMI) and phospholipase D-specific inhibitor, 5-fluoro-2-indolyl des-chlorohalopemide (FIPI) attenuate mercury-induced lipid signaling leading to protection against cytotoxicity in aortic endothelial cells. Int. J. Toxicol..

[49] Böhme M., Diener M., Mestres P., Rummel W. (1992). Direct and indirect actions of HgCl_2_ and methyl mercury chloride on permeability and chloride secretion across the rat colonic mucosa. Toxicol. Appl. Pharmacol..

[50] Lazo J.S., Kuo S.M., Woo E.S., Pitt B.R. (1998). The protein thiol metallothionein as an antioxidant and protectant against antineoplastic drugs. Chem. Biol. Interact..

[51] Nishio H., Nezasa K., Hirano J., Nakata Y. (1996). Effects of thimerosal, an organic sulfhydryl modifying agent, on serotonin transport activity into rabbit blood platelets. Neurochem. Int..

[52] James S.J., Slikker W., Melnyk S., New E., Pogribna M., Jernigan S. (2005). Thimerosal neurotoxicity is associated with glutathione depletion, protection with glutathione precursors. Neurotoxicology.

[53] Anundi I., Högberg J., Stead A.H. (1979). Glutathione depletion in isolated hepatocytes, its relation to lipid peroxidation and cell damage. Acta Pharmacol. Toxicol. (Copenh)..

[54] Agrawal A., Kaushal P., Agrawal S., Gollapudi S., Gupta S. (2007). Thimerosal induces TH2 responses via influencing cytokine secretion by human dendritic cells. J. Leukoc. Biol..

[55] Abdel-Rahman M., Mohamed A.F., Essam N., Moneim A.E.A. (2013). Studies on H1N1 vaccine-induced monoamines alternations and oxidative stress on brain of adult mice. J. Appl. Pharm. Sci..

[56] Stringari J., Meotti F.C., Souza D.O., Santos A.R., Farina M. (2006). Postnatal methylmercury exposure induces hyperlocomotor activity and cerebellar oxidative stress in mice, dependence on the neurodevelopmental period. Neurochem. Res..

[57] Manfroi C.B., Schwalm F.D., Cereser V., Abreu F., Oliveira A., Bizarro L., Rocha J.B., Frizzo M.E., Souza D.O., Farina M. (2004). Maternal milk as methylmercury source for suckling mice, neurotoxic effects involved with the cerebellar glutamatergic system. Toxicol. Sci..

[58] Franco J.L., Teixeira A., Meotti F.C., Ribas C.M., Stringari J., Garcia Pomblum S.C., Moro A.M., Bohrer D., Bairros A.V., Dafre A.L., Santos A.R., Farina M. (2006). Cerebellar thiol status and motor deficit after lactational exposure to methylmercury. Environ. Res..

[59] Wu X., Liang H., O’Hara K.A., Yalowich J.C., Hasinoff B.B. (2008). Thiol-modulated mechanisms of the cytotoxicity of thimerosal and inhibition of DNA topoisomerase II alpha. Chem. Res. Toxicol..

[60] Nabemoto M., Ohsawa K., Nakamura H., Hirabayashi T., Saito T., Okuma Y., Nomura Y., Murayama T. (2005). Reversible activation of secretory phospholipase A2 by sulfhydryl reagents. Arch. Biochem. Biophys..

[61] Hagele T.J., Mazerik J.N., Gregory A., Kaufman B., Magalang U., Kuppusamy M.L., Marsh C.B., Kuppusamy P., Parinandi N.L. (2007). Mercury activates vascular endothelial cell phospholipase D through thiols and oxidative stress. Int. J. Toxicol..

[62] Zieminska E., Toczylowska B., Stafiej A., Lazarewicz J.W. (2010). Low molecular weight thiols reduce thimerosal neurotoxicity *in vitro*, modulation by proteins. Toxicology.

[63] Olczak M., Duszczyk M., Mierzejewski P., Bobrowicz T., Majewska M.D. (2010). Neonatal administration of thimerosal causes persistent changes in mu opioid receptors in the rat brain. Neurochem. Res..

[64] Makani S., Gollapudi S., Yel L., Chiplunkar S., Gupta S. (2002). Biochemical and molecular basis of thimerosal-induced apoptosis in T cells, a major role of mitochondrial pathway. Genes Immun..

[65] Vas J., Monestier M. (2008). Immunology of mercury. Ann. N.Y. Acad. Sci..

[66] Shenker B.J., Mayro J.S., Rooney C., Vitale L., Shapiro I.M. (1993). Immunotoxic effects of mercuric compounds on human lymphocytes and monocytes. IV. Alterations in cellular glutathione content. Immunopharmacol. Immunotoxicol..

[67] Migdal C., Tailhardat M., Courtellemont P., Haftek M., Serres M. (2010). Responsiveness of human monocyte-derived dendritic cells to thimerosal and mercury derivatives. Toxicol. Appl. Pharmacol..

[68] Mian M.F., Kang C., Lee S., Choi J.H., Bae S.S., Kim S.H., Kim Y.H., Ryu S.H., Suh P.G., Kim J.S., Kim E. (2008). Cleavage of focal adhesion kinase is an early marker and modulator of oxidative stress-induced apoptosis. Chem. Biol. Interact..

[69] Liu S.I., Huang C.C., Huang C.J., Wang B.W., Chang P.M., Fang Y.C., Chen W.C., Wang J.L., Lu Y.C., Chu S.T. (2007). Thimerosal-induced apoptosis in human SCM1 gastric cancer cells, activation of p38 MAP kinase and caspase-3 pathways without involvement of [Ca^2+^]i elevation. Toxicol. Sci..

[70] Migdal C., Foggia L., Tailhardat M., Courtellemont P., Haftek M., Serres M. (2010). Sensitization effect of thimerosal is mediated *in vitro* via reactive oxygen species and calcium signaling. Toxicology.

[71] Ueha-Ishibashi T., Tatsuishi T., Iwase K., Umebayashi C., Hirama S., Sakai Y., Ishida S., Okano Y. (2004). Property of thimerosal-induced decrease in cellular content of glutathione in rat thymocytes, a flow cytometric study with 5-chloromethylfluorescein diacetate. Toxicol. in Vitro.

[72] Montero M., Barrero M.J., Torrecilla F., Lobatón C.D., Moreno A., Alvarez J. (2001). Stimulation by thimerosal of histamine-induced Ca(^2+^) release in intact HeLa cells seen with aequorin targeted to the endoplasmic reticulum. Cell Calcium.

[73] Bootman M.D., Taylor C.W., Berridge M.J. (1992). The thiol reagent, thimerosal, evokes Ca^2+^ spikes in HeLa cells by sensitizing the inositol 1,4,5-trisphosphate receptor. J. Biol. Chem..

[74] Waring R.H., O’Reilly B.A. (1990). Enzyme and sulphur oxidation deficiencies in autistic children with known food/chemical intolerances. Xenobiotica.

[75] Alberti A., Pirrone P., Elia M., Waring R.H., Romano C. (1999). Sulphation deficit in low-functioning autistic children, a pilot study. Biol. Psychiatr..

[76] Waring R.H., Klovrza L.V. (2000). Sulphur metabolism in autism. J. Nutr. Environ. Med..

[77] Dawson P.A., Markovich D. (2007). Genetic polymorphisms of human sulfate transporters. Curr. Pharmacogenom..

[78] Markovich D., Mure H., Biber J., Sakhaee K., Pak C., Levi M. (1998). Dietary sulfate regulates the expression of the renal brush border Na/Si cotransporter, NaSi-1. J. Am. Soc. Nephrol..

[79] Blaurock-Busch E., Amin O.R., Dessoki H.H., Rabah T. (2012). Toxic metals and essential elements in hair and severity of symptoms among children with autism. Maedica (Buchar).

[80] Geier D.A., Geier M.R. (2006). A clinical and laboratory evaluation of methionine cycle-transsulfuration and androgen pathway markers in children with autistic disorders. Horm. Res..

[81] Paşca S.P., Dronca E., Kaucsár T., Craciun E.C., Endreffy E., Ferencz B.K., Iftene F., Benga I., Cornean R., Banerjee R., Dronca M.  (2009). One carbon metabolism disturbances and the C677T MTHFR gene polymorphism in children with autism spectrum disorders. J. Cell Mol. Med..

[82] Al-Yafee Y.A., Al-Ayadhi L.Y., Haq S.H., El-Ansary A.K. (2011). Novel metabolic biomarkers related to sulfur-dependent detoxification pathways in autistic patients of Saudi Arabia. BMC Neurol..

[83] Al-Gadani Y., El-Ansary A., Attas O., Al-Ayadhi L. (2009). Metabolic biomarkers related to oxidative stress and antioxidant status in Saudi autistic children. Clin. Biochem..

[84] Geier D.A., Kern J.K., Adams J.B., Garver C.R., Geier M.R. (2009). A prospective study of oxidative stress biomarkers in autistic disorders. J. Appl. Psychol..

[85] Adams J.B., Audhya T., McDonough-Means S., Rubin R.A., Quig D., Geis E., Gehn E., Loresto M., Mitchell J., Atwood S., Barnhouse S., Lee W. (2011). Nutritional and metabolic status of children with autism *vs*. neurotypical children, and the association with autism severity. Nutr. Metab. (Lond)..

[86] Main P.A., Angley M.T., O'Doherty C.E., Thomas P., Fenech M. (2012). The potential role of the antioxidant and detoxification properties of glutathione in autism spectrum disorders: A systematic review and meta-analysis. Nutr. Metab. (Lond)..

[87] Chauhan A., Audhya T., Chauhan V. (2012). Brain region-specific glutathione redox imbalance in autism. Neurochem. Res..

[88] Rose S., Melnyk S., Pavliv O., Bai S., Nick T.G., Frye R.E., James S.J. (2012). Evidence of oxidative damage and inflammation associated with low glutathione redox status in the autism brain. Transl. Psychiatry.

[89] Dringen R., Pfeiffer B., Hamprecht B. (1999). Synthesis of the antioxidant glutathione in neurons: Supply by astrocytes of CysGly as precursor for neuronal glutathione. J. Neurosci..

[90] Bounous G., Molson J. (1999). Competition for glutathione precursors between the immune system and the skeletal muscle, pathogenesis of chronic fatigue syndrome. Med. Hypotheses.

[91] Ho W.Z., Douglas S.D. (1992). Glutathione and N-acetylcysteine suppression of human immunodeficiency virus replication in human monocyte/macrophages *in vitro*. AIDS Res. Hum. Retrovir..

[92] Sen C.K. (1997). Nutritional biochemistry of cellular glutathione. J. Nutr. Biochem..

[93] Baruchel S., Viau G., Olive R., Bounous G., Montagnier L., Olivier R., Pasquier C. (1998). Nutraceutical Modulation with a Humanized Native Milk Serum Protein Isolate, Immunocal®, Application in AIDS and Cancer. Oxidative Stress in Cancer, AIDS and Neurodegenerative Diseases.

[94] Gutman J. (2002). Glutathione—Your Bodies Most Powerful Protector.

[95] Takahashi T., Kimura T., Sato Y., Shiraki H., Ukita T. (1971). Time-dependent distribution of [203] Hg-mercury compounds in rat and monkey as studied by whole body autoradiography. J. Hygenic. Chem. (Japan).

[96] Geier D.A., Kern J.K., Adams J.B., Geier M.R. (2009). Biomarkers of environmental toxicity and susceptibility in autism. J. Neurolog. Sci..

[97] Geier D.A., Kern J.K., Garver C.R., Adams J.B., Audhya T., Geier M.R. (2009). A prospective study of transsulfuration biomarkers in autistic disorders. Neurochem. Res..

[98] Nkabyo Y.S., Gu L.H., Jones D.P., Ziegler T.R. (2006). Thiol/disulfide redox status is oxidized in plasma and small intestinal and colonic mucosa of rats with inadequate sulfur amino acid intake. J. Nutr..

[99] Sharpe M.A., Taylor L., Gist T.L., Baskin D.S. (2013). B-Lymphocytes from a population of children with autism spectrum disorder and their unaffected siblings exhibit hypersensitivity to Thimerosal. J. Toxicol..

[100] Bigham M., Copes R. (2005). Thiomersal in vaccines, balancing the risk of adverse effects with the risk of vaccine-preventable disease. Drug Saf..

[101] Dórea J.G., Bezerra V.L., Fajon V., Horvat M. (2011). Speciation of methyl- and ethyl-mercury in hair of breastfed infants acutely exposed to thimerosal-containing vaccines. Clin. Chim. Acta.

[102] Dórea J.G., Marques R.C., Isejima C. (2012). Neurodevelopment of Amazonian infants, antenatal and postnatal exposure to methyl- and ethylmercury. J. Biomed. Biotechnol..

[103] Redwood L., Bernard S., Brown D. (2001). Predicted mercury concentrations in hair from infant immunizations, cause for concern. Neurotoxicology.

[104] El-baz F., Elhossiny E.M., Elsayed A.B., Gaber G.M. (2010). Hair mercury measurement in Egyptian autistic children. Egyptian J. Med. Hum. Genet..

[105] Majewska M.D., Urbanowicz E., Rok-Bujko P., Namyslowska I., Mierzejewski P.  (2010). Age-dependent lower or higher levels of hair mercury in autistic children than in healthy controls. Acta Neurobiol. Exp. (Wars).

[106] Jedrychowski W., Jankowski J., Flak E., Skarupa A., Mroz E., Sochacka-Tatara E., Lisowska-Miszczyk I., Szpanowska-Wohn A., Rauh V., Skolicki Z. (2006). Effects of prenatal exposure to mercury on cognitive and psychomotor function in one-year-old infants, epidemiologic cohort study in Poland. Ann. Epidemiol..

[107] Lederman S.A., Jones R.L., Caldwell K.L., Rauh V., Sheets S.E., Tang D., Viswanathan S., Becker M., Stein J.L., Wang R.Y., Perera F.P. (2008). Relation between cord blood mercury levels and early child development in a World Trade Center cohort. Environ. Health Perspect..

[108] Björnberg K.A., Vahter M., Petersson-Grawé K., Glynn A., Cnattingius S., Darnerud P.O., Atuma S., Aune M., Becker W., Berglund M. (2003). Methyl mercury and inorganic mercury in Swedish pregnant women and in cord blood, influence of fish consumption. Environ. Health Perspect..

[109] Ouédraogo O., Amyot M. (2011). Effects of various cookingmethods and foodcomponents on bioaccessibility of mercury from fish. Environ. Res..

[110] Burbacher T.M., Shen D.D., Liberato N., Grant K.S., Cernichiari E., Clarkson T. (2005). Comparison of blood and brain mercury levels in infant monkeys exposed to methylmercury or vaccines containing thimerosal. Environ. Health Perspect..

[111] World Health Organization Mercury Intoxication. http://www.paho.org/hq/index.php?option=com_content&view=article&id=8158&Itemid=39767&lang=en.

[112] Pichichero M.E., Gentile A., Giglio N., Umido V., Clarkson T., Cernichiari E., Zareba G., Gotelli C., Gotelli M., Yan L., Treanor J. (2008). Mercury levels in newborns and infants after receipt of thimerosal-containing vaccines. Pediatrics.

[113] Takeda Y., Kunugi T., Hoshino O., Ukita T. (1968). Distribution of inorganic, aryl, and alkyl mercury compounds in rats. Toxicol. Appl. Pharmacol..

[114] Zimmer B., Lee G., Balmer N.V., Meganathan K., Sachinidis A., Studer L., Leist M. (2012). Evaluation of developmental toxicants and signaling pathways in a functional test based on the migration of human neural crest cells. Environ. Health Perspect..

[115] Ueha-Ishibashi T., Oyama Y., Nakao H., Umebayashi C., Nishizaki Y., Tatsuishi T., Iwase K., Murao K., Seo H. (2004). Effect of thimerosal, a preservative in vaccines, on intracellular Ca^2+^ concentration of rat cerebellar neurons. Toxicology.

[116] Zimmermann L.T., Santos D.B., Naime A.A., Leal R.B., Dórea J.G., Barbosa F., Aschner M., Rocha J.B., Farina M. (2013). Comparative study on methyl- and ethylmercury-induced toxicity in C6 glioma cells and the potential role of LAT-1 in mediating mercurial-thiol complexes uptake. Neurotoxicology.

[117] Peltz A., Sherwani S.I., Kotha S.R., Mazerik J.N., O'Connor Butler E.S., Kuppusamy M.L., Hagele T., Magalang U.J., Kuppusamy P., Marsh C.B., Parinandi N.L. (2009). Calcium and calmodulin regulate mercury-induced phospholipase D activation in vascular endothelial cells. Int. J. Toxicol..

[118] Olczak M., Duszczyk M., Mierzejewski P., Wierzba-Bobrowicz T., Majewska M.D. (2010). Lasting neuropathological changes in rat brain after intermittent neonatal administration of thimerosal. Folia Neuropathol..

[119] Hargreaves R.J., Eley B.P., Moorhouse S.R., Pelling D. (1988). Regional cerebral glucose metabolism and blood flow during the silent phase of methylmercury neurotoxicity in rats. J. Neurochem..

[120] Nelson K.B., Bauman M.L. (2003). Thimerosal and autism?. Pediatrics.

[121] Dórea J.G., Farina M., Rocha J.B. (2013). Toxicity of ethylmercury (and Thimerosal): A comparison with methylmercury. J. Appl. Toxicol..

[122] Korbas M., O’Donoghue J.L., Watson G.E., Pickering I.J., Singh S.P., Myers G.J., Clarkson T.W., George G.N. (2010). The chemical nature of mercury in human brain following poisoning or environmental exposure. ACS Chem. Neurosci..

[123] U. S. Food and Drug Administration Vaccines, Blood & Biologics. Thimerosal in Vaccines. http://www.fda.gov/BiologicsBloodVaccines/SafetyAvailability/VaccineSafety/UCM096228.

[124] World Health Organization (2006). WHO Statement on Thiomersal.

[125] Geier D.A., Sykes L.K., Geier M.R. (2007). A review of Thimerosal (Merthiolate) and its ethylmercury breakdown product: Specific historical considerations regarding safety and effectiveness. J. Toxicol. Environ. Health B Crit. Rev..

[126] Migliarini S., Pacini G., Pelosi B., Lunardi G., Pasqualetti M. (2012). Lack of brain serotonin affects postnatal development and serotonergic neuronal circuitry formation. Mol. Psychiatry.

[127] LeBlanc J.J., Fagiolini M. (2011). Autism, a “critical period” disorder?. Neural Plast..

[128] Makri A., Goveia M., Balbus J., Parkin R. (2004). Children’s susceptibility to chemicals, a review by developmental stage. J. Toxicol. Environ. Health B.

[129] Graeter L.J., Mortensen M.E. (1996). Kids are different, developmental variability in toxicology. Toxicology.

[130] Ballatori N., Clarkson T.W. (1982). Developmental changes in the biliary excretion of methylmercury and glutathione. Science.

[131] Blackburn S.T. (1994). Renal function in the neonate. J. Perinat. Neonatal Nurs..

[132] Ida-Eto M., Oyabu A., Ohkawara T., Tashiro Y., Narita N., Narita M. (2013). Prenatal exposure to organomercury, thimerosal, persistently impairs the serotonergic and dopaminergic systems in the rat brain: implications for association with developmental disorders. Brain Dev..

[133] Palmer R.F., Banchard S., Stein Z., Mandell D., Miller C. (2006). Environmental mercury release, special education rates, and autistic disorder, an ecological study of Texas. Health Place.

[134] Palmer R.F., Blanchard S., Wood R. (2009). Proximity to point sources of environmental mercury release as a predictor of autism prevalence. Health Place.

[135] Windham G.C., Zhang L., Gunier R., Croen L.A., Grether J.K. (2006). Autism spectrum disorders in relation to distribution of hazardous air pollutants in the San Francisco Bay area. Environ. Health Perspect..

[136] Young H.A., Geier D.A., Geier M.R. (2008). Thimerosal exposure in infants and neurodevelopmental disorders, an assessment of computerized medical records in the Vaccine Safety Datalink. J. Neurol. Sci..

[137] Geier D.A., Geier M.R. (2007). A prospective study of mercury toxicity biomarkers in autistic spectrum disorders. J. Toxicol. Environ. Health.

[138] Geier D.A., Mumper E., Gladfelter B., Coleman L., Geier M.R. (2008). Neurodevelopmental disorders, maternal Rh-negativity, and Rho(D) immune globulins, a multi-center assessment. Neuro. Endocrinol. Lett..

[139] Geier D.A., Kern J.K., King P.G., Sykes L.K., Geier M.R. (2012). Hair toxic metal concentrations and autism spectrum disorder severity in young children. Int. J. Environ. Res. Public Health.

[140] Adams J.B., Baral M., Geis E., Mitchell J., Ingram J., Hensley A., Zappia I., Newmark S., Gehn E., Rubin R.A., Mitchell K., Bradstreet J., El-Dahr J. (2009). Safety and efficacy of oral DMSA therapy for children with autism spectrum disorders, part A–Medical results. BMC Clin. Pharmacol..

[141] Blanchard K.S., Palmer R.F., Stein Z. (2011). The value of ecologic studies, mercury concentration in ambient air and the risk of autism. Rev. Environ. Health.

[142] Elshenshtawy E., Tobar S., Sherra K., Atallah S., Elkasaby R. (2011). Study of some biomarkers in hair of children with autism. Middle East Curr. Psychiatry.

[143] Lakshmi Priya M.D., Geetha A. (2011). Level of trace elements (copper, zinc, magnesium and selenium) and toxic elements (lead and mercury) in the hair and nail of children with autism. Biol. Trace Elem. Res..

[144] Sajdel-Sulkowska E.M., Lipinsk B., Windom H., Audhya T., McGinnis W. (2008). Oxidative stress in autism, elevated cerebellar 3-nitrotyrosine levels. Am. J. Biochem. Biotechnol..

[145] Kern J.K., Geier D.A., Adams J.B., Grannemann B.D., Mehta J.A., Geier M.R. (2011). Toxicity biomarkers related to autism spectrum disorder, a blinded study of urinary porphyrins. Pediatr. Int..

[146] Kern J.K., Geier D.A., Audhya T., King P.G., Sykes L., Geier M. (2012). Evidence of parallels between mercury intoxication and the brain pathology in autism. Acta Neurobiol. Exp. (Warsz).

[147] Geier D.A., Audhya T., Kern J.K., Geier M.R. (2010). Differences in blood mercury levels in autism spectrum disorders, is there a threshold level?. Acta Neurobiol. Exp..

[148] Kern J.K., Geier D.A., Adams J.B., Geier M.R. (2010). A Biomarker of mercury body-burden correlated with diagnostic domain specific clinical symptoms of autistic disorders. Biometals..

[149] Nataf R., Skorupka C., Amet L., Lam A., Springbett A., Lathe R. (2006). Porphyinuria in childhood autistic disorder, implications for environmental toxicity. Toxicol. Applied Pharmacol..

[150] Holmes A.S., Blaxill M.F., Haley B.E. (2003). Reduced levels of mercury in first baby haircuts of autistic children. Int. J. Toxicol..

[151] Ida-Eto M., Oyabu A., Ohkawara T., Tashiro Y., Narita N., Narita M. (2011). Embryonic exposure to thimerosal, an organomercury compound, causes abnormal early development of serotonergic neurons. Neuropharmacology.

[152] Azmitia E.C., Singh J.S., Whitaker-Azmitia P.M. (2011). Increased serotonin axons (immunoreactive to 5-HT transporter) in postmortem brains from young autism donors. Neurosci. Lett..

[153] Mrozek-Budzyn D., Majewska R., Kieltyka A., Augustyniak M. (2012). Neonatal exposure to Thimerosal from vaccines and child development in the first 3 years of life. Neurotoxicol. Teratol..

[154] Thompson W.W., Price C., Goodson B., Shay D.K., Benson P., Hinrichsen V.L., Lewis E., Eriksen E., Ray P., Marcy S.M. (2007). Early thimerosal exposure and neuropsychological outcomes at 7 to 10 years. Vaccine Safety Datalink Team. N. Engl. J. Med..

[155] Verstraeten T., Davis R.L., DeStefano F., Lieu T.A., Rhodes P.H., Black S.B., Shinefield H., Chen R.T., Vaccine Safety Datalink Team (2003). Safety of thimerosal-containing vaccines, a two-phased study of computerized health maintenance organization databases. Pediatrics.

[156] Andrews N., Miller E., Grant A., Stowe J., Osborne V., Taylor B. (2004). Thimerosal exposure in infants and developmental disorders, a retrospective cohort study in the United Kingdom does not support a causal association. Pediatrics.

[157] Barile J.P., Kuperminc G.P., Weintraub E.S., Mink J.W., Thompson W.W. (2012). Thimerosal exposure in early life and neuropsychological outcomes 7–10 years later. J. Pediatr. Psychol..

[158] Geier D.A., Geier M.R. (2005). A two-phased population epidemiological study of the safety of thimerosal-containing vaccines, a follow-up analysis. Med. Sci. Monit..

[159] Moro P.L., Broder K., Zheteyeva Y., Revzina N., Tepper N., Kissin D., Barash F., Arana J., Brantley M.D., Ding H. (2011). Adverse events following administration to pregnant women of influenza A (H1N1) 2009 monovalent vaccine reported to the Vaccine Adverse Event Reporting System. Am. J. Obstet. Gynecol..

[160] Geier D.A., Young H.A., Geier M.R. (2010). Thimerosal exposure & increasing trends of premature puberty in the vaccine safety datalink. Indian J. Med. Res..

[161] Balabanič D., Rupnik M., Klemenčič A.K. (2011). Negative impact of endocrine-disrupting compounds on human reproductive health. Reprod. Fertil. Dev..

[162] Hotchkiss A.K., Rider C.V., Blystone C.R., Wilson V.S., Hartig P.C., Ankley G.T., Foster P.M., Gray C.L., Gray L.E. (2008). Fifteen years after “Wingspread”—Environmental endocrine disrupters and human and wildlife health: Where we are today and where we need to go. Toxicol. Sci..

[163] Tan S.W., Meiller J.C., Mahaffey K.R. (2009). The endocrine effects of mercury in humans and wildlife. Crit. Rev. Toxicol..

[164] Fagan D.G., Pritchard J.S., Clarkson T.W., Greenwood M.R. (1977). Organ mercury levels in infants with omphaloceles treated with organic mercurial antiseptic. Arch. Dis. Child.

[165] Barcelos G.R., Grotto D., de Marco K.C., Valentini J., Lengert A.V., Oliveira A.A., Garcia S.C., Braga G.U., Schläwicke Engström K., Cólus I.M. (2013). Polymorphisms in glutathione-related genes modify mercury concentrations and antioxidant status in subjects environmentally exposed to methylmercury. Sci. Total Environ..

[166] Blumberg S.J., Bramlett M.D., Kogan M.D., Schieve L.A., Jones J.R., Lu M.C. Changes in Prevalence of Parent-Reported Autism Spectrum Disorder in School-Aged U.S. Children, 2007 to 2011–2012. http://www.cdc.gov/nchs/data/nhsr/nhsr065.pdf.

[167] Boyle C.A., Boulet S., Schieve L.A., Cohen R.A., Blumberg S.J., Yeargin-Allsopp M., Visser S., Kogan M.D. (2011). Trends in the prevalence of developmental disabilities in US children, 1997–2008. Pediatrics.

